# Phosphorylation Variation during the Cell Cycle Scales with Structural Propensities of Proteins

**DOI:** 10.1371/journal.pcbi.1002842

**Published:** 2013-01-10

**Authors:** Stefka Tyanova, Jürgen Cox, Jesper Olsen, Matthias Mann, Dmitrij Frishman

**Affiliations:** 1 Genome-Oriented Bioinformatics Department, Technische Universität München, Freising, Germany; 2 Department for Proteomics and Signal Transduction, Max-Planck Institute of Biochemistry, Martinsried, Germany; 3 Department of Proteomics, Novo Nordisk Foundation Center for Protein Research, University of Copenhagen, Copenhagen, Denmark; 4 Helmholtz Center Munich - German Research Center for Environmental Health (GmbH), Institute of Bioinformatics and Systems Biology, Neuherberg, Germany; University of California San Diego, United States of America

## Abstract

Phosphorylation at specific residues can activate a protein, lead to its localization to particular compartments, be a trigger for protein degradation and fulfill many other biological functions. Protein phosphorylation is increasingly being studied at a large scale and in a quantitative manner that includes a temporal dimension. By contrast, structural properties of identified phosphorylation sites have so far been investigated in a static, non-quantitative way. Here we combine for the first time dynamic properties of the phosphoproteome with protein structural features. At six time points of the cell division cycle we investigate how the variation of the amount of phosphorylation correlates with the protein structure in the vicinity of the modified site. We find two distinct phosphorylation site groups: intrinsically disordered regions tend to contain sites with dynamically varying levels, whereas regions with predominantly regular secondary structures retain more constant phosphorylation levels. The two groups show preferences for different amino acids in their kinase recognition motifs - proline and other disorder-associated residues are enriched in the former group and charged residues in the latter. Furthermore, these preferences scale with the degree of disorderedness, from regular to irregular and to disordered structures. Our results suggest that the structural organization of the region in which a phosphorylation site resides may serve as an additional control mechanism. They also imply that phosphorylation sites are associated with different time scales that serve different functional needs.

## Author Summary

Cells employ protein phosphorylation – the addition of a phosphate group to serine, threonine or tyrosine residues – as a key regulatory mechanism for modulating protein function. Proteomics technologies can now quantify thousands of phosphorylation sites to reveal the dynamics of phosphorylation at each site in response to a biological process. It is known that phosphorylation does not occur randomly with regard to a protein's structure, but so far the relationship between the dynamics of phosphorylation and these structural properties has not been investigated. Here we relate the relative levels of phosphorylation for more than 5,000 sites through the cell cycle to the predicted structural features of the vicinity of the sites. We find that dynamic phosphorylation tends to occur in disordered regions, whereas phosphorylation sites that did not vary as much over the cell cycle are often located in defined secondary structure elements. Kinases that prefer charged amino acids in their substrate motives are more often associated with unchanging sites whereas proline-directed protein kinases phosphorylate cell cycle regulated sites in disordered regions more frequently. The structural organization of the region in which a phosphorylation site resides may therefore serve as an additional control mechanism in kinase mediated regulation.

## Introduction

Phosphorylation is a ubiquitous post-translational modification that is known to be important for the regulation of a myriad of cellular processes, among which are cell growth, apoptosis, differentiation, signal transduction and transport [1]. Rapidly evolving mass spectrometry (MS)-based technologies, innovative labeling techniques and advances in computational proteomics provide powerful means for overcoming the low abundance problem of this modification and are making it possible to obtain large-scale, high-resolution quantitative data. With these advances, not only can single protein phosphorylation experiments be done with high accuracy, but also whole-phosphoproteome studies are becoming increasingly feasible [2], [3].

Given the availability of these data, much research has been devoted to analyzing and understanding the structural features of phospho-sites. This includes creation of online resources containing structural information [4], combining data on linear motifs and structural properties [5], and development of software tools that use three-dimensional data for the prediction of phosphorylation sites (DISPHOS [6], Phos3D [7]). Large-scale studies of the structural characteristics of phosphorylation sites have focused on solvent exposure, local and global structure, amino acid context of the spatial surrounding, and structural motifs [7]–[9]. The mechanism of modification suggests that serine, threonine and tyrosine residues should be located on the protein surface where they are accessible for the modifying kinase [7].

The main challenge in studying structural properties of phospho-sites from experimental data is their preference for unstructured regions [6] for which electron density is often missing in X-ray structures. Disorder is strongly associated with protein-protein interactions [10]. Modified residues found within disordered regions can act as on/off switches, either promoting or inhibiting an interaction. Due to the specific structural organization of some protein kinases, in which the catalytic loop resides within a small cleft between two lobes, flexible regions within the substrate's interaction surface are well suited for binding to the kinase. However, a recent systematic study suggested that kinase preference for disordered regions is only marginal [8]. Furthermore, a computational study of kinase specificity reported that approximately 60% of the sites modified by protein kinase A lie within α-helical regions [11]. These considerations raise an interesting question: can a distinction between kinases be made with respect to the level of structural organization of their substrates.

Since phosphorylation events both depend on the structural environment and influence its properties, protein structure and phosphorylation should be considered interrelated and mutually dependent. On one hand, disorder facilitates the access of a kinase to the residue to be modified. On the other hand, the addition of a phosphate moiety may lead to structural changes. Both order-to-disorder and disorder-to-order transitions upon phosphorylation have been observed in nature or studied via molecular dynamics simulations [12]–[15]. The major driving forces of conformational changes observed upon phosphorylation are the electrostatic interactions between the negatively charged phosphate group and the surrounding charged residues. The functional roles of charged residues range from stabilization to correct substrate identification and facilitation of conformational changes.

Although numerous previous studies have focused on structural properties of phosphorylation sites [6]–[8] no systematic analysis has been performed combining large-scale quantitative data with structural features. To bridge this gap we here build on data from a recent study by Olsen *et al.*, which elucidated phosphorylation site occupancy during mitosis [16]. Quantitative data were measured at six time points, corresponding to major phases of the cell division cycle. The additional temporal dimension of these data makes it possible to examine how various phosphorylation sites are dynamically regulated. Olsen *et al.* clustered sites according to their distinct phosphorylation patterns and similarities in regulation with the aim to infer each site's functional importance. Here, in contrast, we focus on structural properties of the phosphorylation sites and, for the first time, distinguish between two groups of sites with respect to the overall variation of phosphorylation over time.

We find that sites that lie within regular secondary structures exhibit less variable phosphorylation fold changes during the cell cycle than sites that are found in disordered regions. Analysis of the amino acid composition of the flanking regions of these two groups of sites revealed enrichment of positively charged residues and depletion of disorder-related residues such as proline, serine and threonine in the former group.

## Results

### Sites within disordered regions exhibit larger variation of phosphorylation

Using the data from the Olsen *et al.* investigation [16], we here computed the overall variation of the phosphorylation ratios during six time points of the cell cycle and investigated the differences between the sites with small variation as opposed to the sites with large variation. The original data set comprised 6,027 proteins with 20,443 unique phosphorylation sites. We retained only those sites that had quantitative information for all six time points available (1,059 proteins with 5,173 sites). The phospho-site variability is calculated as the standard deviation of the phosphorylation ratios over the six time points measured during the cell cycle.

**Figure 1:**
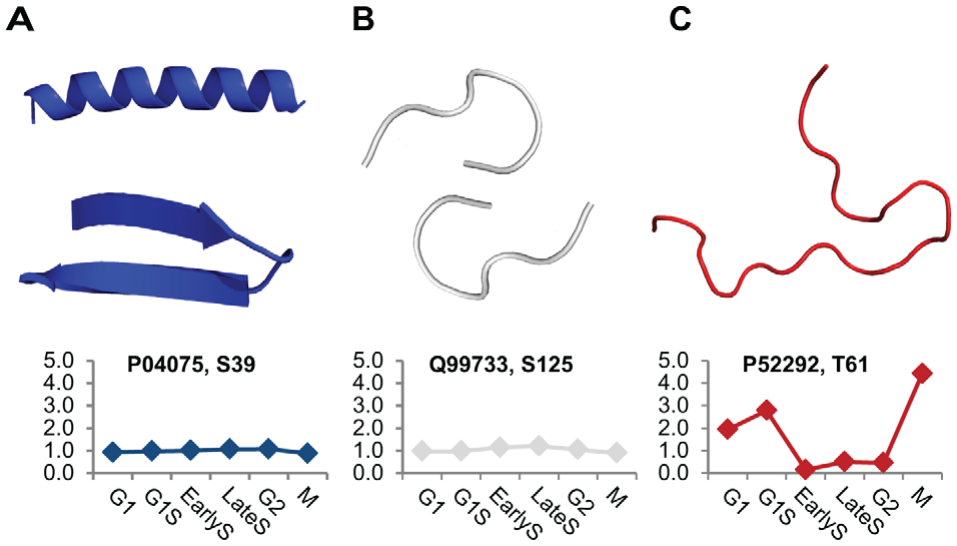
Temporal phosphorylation patterns of phospho-sites with distinct structural properties. Phosphorylation fold changes of three sites (UniProt accession number and residue identification number are given) during the six time points is shown together with their corresponding local structure. From left to right the phosphorylation variation over the six time points increases, together with the level of disorder: from (**A**) regular secondary structure (α-helix or β-sheet) through (**B**) irregular coils and loops to (**C**) disordered regions. Phospho-serine residues (pS) within regular regions and loops show small fluctuations in their phosphorylation levels, while larger changes occur in disordered regions.

We sought to investigate a possible relation between the structural organization of the environment in which a modified residue is found and the experimentally measured changes in phosphorylation during the cell cycle. To do so, we compared the phosphorylation variation of two groups of sites. These two groups were composed of sites that reside in ordered regions and sites that lie within disordered regions as predicted with DISOPRED [17]. In agreement with previously observed tendency we found over 90% of the modified residues to lie within disordered regions (4,675 sites versus 498 sites). Figure 1 shows three examples from our large-scale dataset, illustrating a non-variable site on a regular secondary structure (α-helix), a slightly variable site on a short loop and a variable site in a disordered region.

**Figure 2:**
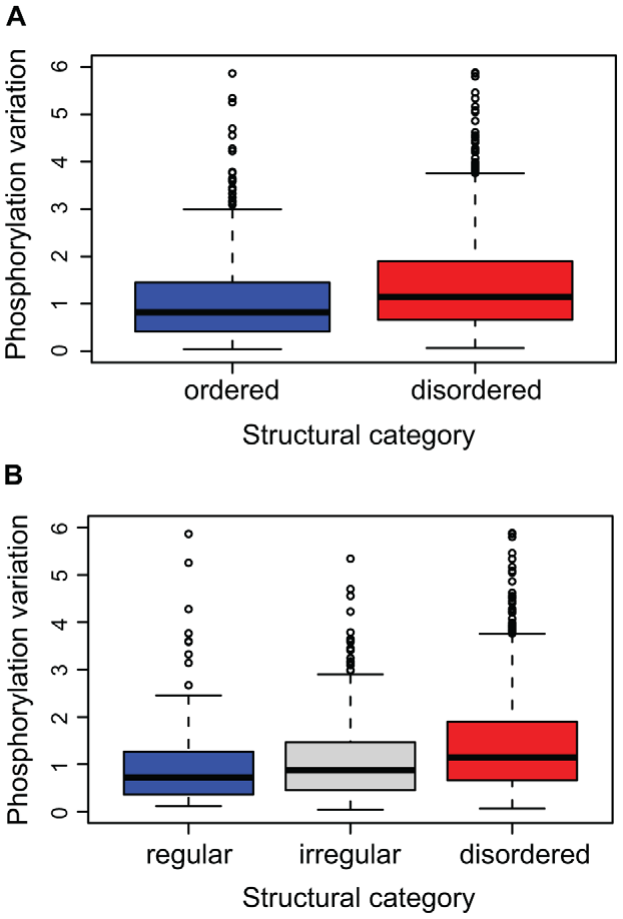
Comparison of phosphorylation variation of sites within different structural categories. **A**) Sites within ordered regions (blue) show smaller variation of the phosphorylation fold change over the cell cycle than those within disordered regions (red). The significance of the observation has been tested with Kolmogorov-Smirnov test (p-value 6.6E-13). (**B**) The variation of phosphorylation changes over the cell cycle scales with the structural propensities of the phosphorylated residues: from lowest in regular structures (blue) to highest in disordered regions (red). The observed differences were found to be significant by ANOVA test (p-value 3.02E-09).

Our results revealed notable differences in the distributions of phosphorylation variations of the two sets (Kolmogorov-Smirnov test p-value 6.6E-13). The sites associated with structurally characterized regions were found to exhibit smaller changes in phosphorylation during the cell cycle (median 1.77) as compared to sites located in disordered regions (median 2.22, Figure 2A).

### Phosphorylation variability scales with the level of structural order

Having investigated the difference between ordered and disordered regions on a global scale, we next predicted protein secondary structure in more detail using PsiPred [18]. First we classified sites into regular structures (92 in α-helices or 53 in β-sheets) and sites with irregular structures (5,028 in loops, turns and coils). Phosphorylation in regular secondary structures showed smaller variation over the six time points of the cell cycle. This effect was small but statistically significant (ANOVA p-value 1.8E-04).

Although there is a large intersection between ordered structures and regular secondary structures, and the terms are often used interchangeably, the two sets are not identical. We observed that a large number of regions predicted as ‘*coil*’ by PsiPred are predicted as ‘*ordered*’ by DISOPRED. This reflects a distinction between ordered and disordered coils. A major difference between these two groups of coils is the length distribution of their elements (p-value 4.12E-114): ‘ordered coils’ are much shorter on average as they mainly correspond to turns and short loops connecting regular secondary structures. By contrast, ‘disordered regions’ are longer and represent large protein regions lacking defined structure (see Text S1 for details).

In order to take this distinction into account, we redefined the structural environments into three categories: regular structures (predicted as helix/sheet and ordered), irregular structures (predicted as coil and ordered), and disordered regions (predicted as coil and disordered) (Figure 2B). We found significant differences in the variation of the phosphorylation ratios between these distinct structural groups (ANOVA p-value 3.02E-09). Sites within ordered structural environments appeared to be subjected to the lowest level of regulation during the cell cycle (median 1.65). Interestingly, a distinction emerged between coils (median 1.83) and disordered regions (median 2.22), signifying that the latter exhibited the largest variation in phosphorylation changes. We speculate that the increased variation of phosphorylation in longer, disordered coils correlates with their higher solvent exposure, which makes them more easily accessible for both kinases and phosphatases. Overall, our data shows that the phosphorylation variation of a site clearly scales with the level of order of its structural context (i.e. the tendency of a site to be found within a regular, irregular or disordered region).

### Amino acid content of flanking regions of sites with different phosphorylation variation

**Figure 3:**
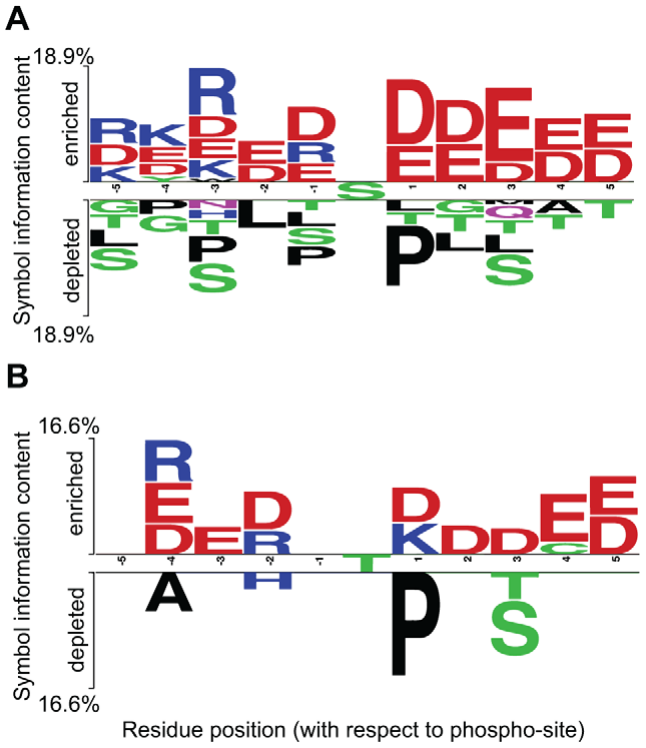
Two sample logo of flanking regions of phosphorylation sites of low *versus* high phosphorylation variation. Amino acids in the top and bottom parts (**A**) – central residue serine and **B**) – central residue threonine) represent residues, which are enriched or depleted correspondingly in the flanking regions of sites with small phosphorylation variation. Strong preferences are found for charged residues such as arginine, aspartate, and glutamate. In contrast, the majority of the amino acids that are more frequent in the negative set (i.e. variable phosphorylation set) are disorder-related e.g. proline, serine and glycine.

We wanted to investigate if sites with distinct phosphorylation patterns over the cell cycle differ not only according to structural context, but also with respect to the amino acid content in their local sequence environment. A two-sample logo [19] was computed to contrast the two data sets, using the highly variable sites as a negative set (Figure 3). For each position and each possible amino acid, a two sample t-test was used to evaluate the null-hypothesis that the vectors of residues at a given position in both the positive and negative data sets (*i.e.* low and high variation) come from the same distribution. We found statistically significant enrichment of charged amino acids and depletion of proline, serine and threonine in the surrounding of sites with small phosphorylation variability (p-value<0.05). Additional comparisons of the amino acid distributions of the two sets against a background distribution accounting for structural differences using the composition profiling technique [20] revealed similar trends (see Text S1 for the detailed analysis and results).

The enrichment of serine and threonine residues in the vicinity of the detected phosphorylation sites could correlate with additional modification events. To check this hypothesis, we determined if multiple phosphorylation sites are found with higher preference in disordered regions. Phosphorylation sites that had at least one neighboring phosphorylation site in both ordered and disordered regions were compared. A ‘neighbor’ was defined as any phosphorylated residue that lies within +/−1,2,3,4, or 5 residue-long flanking region of a given modification site. Regardless of which of these five cut-offs was chosen, multiple phosphorylation sites were always highly significantly enriched in disordered regions (Table 1).

**Table 1:** Enrichment of multi-phospho sites in disordered regions.

Distance +/−	odds ratio	P-value
1	1.45	8.15E-04
2	1.64	1.80E-07
3	1.69	6.86E-08
4	1.90	3.72E-10
5	1.96	2.36E-10

The enrichment of additional phosphorylation sites at different distances from the central modified residues was computed. The odds ratios were calculated with the Fisher's Exact test implemented in R. Multiple phosphorylation sites were found significantly more often in disordered regions for any of the considered distances.

### Evolutionary analysis of phosphorylation sites with different structural backgrounds

Next, we were interested in potential differences in evolutionary constraints on the phospho-sites in structured and disordered regions. When analyzing conservation it is important to take into account the different evolutionary rates of disordered and ordered regions. We therefore compared conservation scores between phosphorylated serines, threonines and tyrosines with ‘control’ serine, threonine and tyrosine residues with a similar structural background. We define the set of ‘control’ residues, as all potential phosphorylation sites that were not found to be phosphorylated in the study of Olsen *et al.*

**Figure 4:**
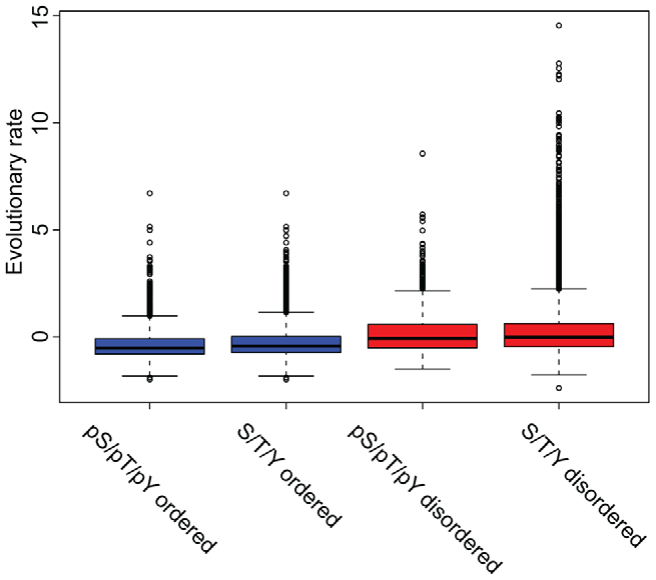
Conservation of phosphorylated sites versus conservation of control sites taking into account local structure. Lower values correspond to slower evolutionary rate and higher conservation. Phosphorylation sites predicted to lie within regular structures (in blue, pS/pT/pY regular) appeared to be more conserved than their equivalent non-phosphorylated residues from the same proteins (p-value 2.24e-16). The same tendency was present for modified sites in disordered regions (in red, pS/pT/pY disordered), which were also subjected to a statistically significant slower evolutionary rate than their control set (p-value 3.4E-03). Phosphorylation sites in regular structures showed higher conservation than that of phosphorylation sites in irregular structures (p-value 3.23E-120).

As expected, phospho-sites that were predicted to lie in regular regions appeared significantly more conserved than phospho-sites in disordered regions (p-value 3.23E-120), due to the more conserved structural background of the former (Figure 4). In agreement with a previous study [21] modified residues in regions that lack defined structure were more conserved than the control serine, threonine and tyrosine residues with the same surrounding environment (Mann-Whitney Wilcoxon test p-value 3.4E-03). The same holds true for phospho-serine, phospho-threonine and phospho-tyrosine in ordered regions as compared to their equivalent control sets (p-value 2.24E-16). Despite the small size of the effect (groups' means −0.38, −0.28, 0.14 and 0.22 for pS/pT/pY ordered, S/T/Y ordered, pS/pT/pY disordered and S/T/Y disordered respectively) the higher evolutionary pressure on phosphorylated residues suggests functional importance of these sites in a broad range of species.

### Motif decomposition with 2D Annotation Enrichment technique

**Figure 5:**
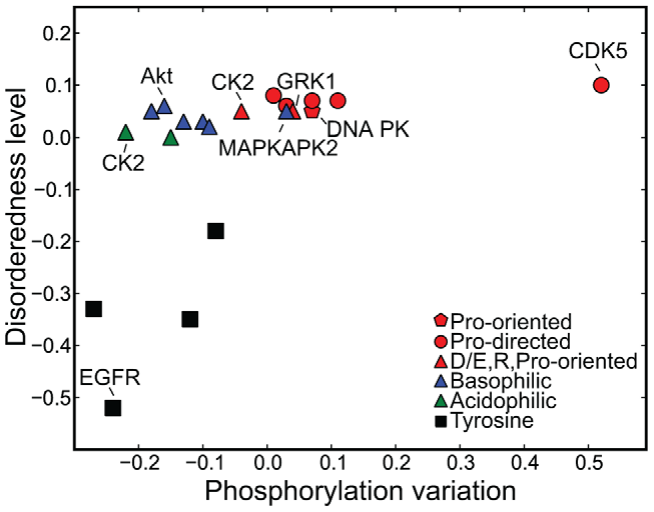
Kinase motif decomposition based on phosphorylation variability and structural preferences. The preferences of various kinases for sites with specific structural background and phosphorylation variation were calculated by the 2D Annotation Enrichment technique (see Methods). In general four classes can be distinguished: (i) tyrosine kinases (black squares), (ii) proline-directed kinases (red circles), (iii) non-proline directed kinases with charged residues in their substrate recognition motif (green and blue triangles corresponding to acidophilic and basophilic kinases respectively) and (iv) proline-oriented kinases, which contain a proline residue in their motif at position different from +1 relative to the modification site (red triangles and pentagons). The vertical axis separates the kinases according to their structural preferences. Tyrosine kinases favor sites within ordered regions with small phosphorylation variability. Serine/threonine kinases prefer more disordered regions, but span a larger space of phosphorylation variation. There is a tendency towards increasing disorderedness with higher phosphorylation variation, which clearly separates non proline-directed, proline-oriented and proline-directed kinases, the latter being characterized by the largest variation in phosphorylation. Examples for each class are shown and the data of all kinases can be found in Table 2.

We next asked if different groups of kinases would exhibit preferences for less variable or highly variable phosphorylation sites. To identify kinase recognition motifs that show similar behavior with respect to two variables – protein disorder and phosphorylation variation, we used the recently described 2D Annotation Enrichment technique (see Methods and [22]). It employs a two dimensional generalization of the nonparametric two-sample test to detect preferences of a certain group of elements for two numerical attributes simultaneously relative to all other elements. The motifs separation is plotted in Figure 5 (the complete data are available in Table 2). The general trend between disorder and phosphorylation variation is reflected in the plot as sites with more disordered background show also higher variability. For individual kinases a very clear separation reflecting their preference for specific amino acids in their consensus motifs becomes apparent. Overall, four classes can be distinguished: (i) tyrosine kinases (black squares), (ii) proline-directed kinases (red circles), (iii) non-proline directed kinases with charged residues in their substrate recognition motif (green and blue triangles) and (iv) proline-oriented kinases, which contain a proline residue in their motif (red triangles and pentagons).

**Table 2:** Enrichment of kinase recognition motifs with specific preferences for disorder and phosphorylation variation.

Variation	Disorder	Benj. Hoch. FDR	Names
−0.16	0.06	3.20E-03	Akt kinase
−0.27	−0.33	7.67E-05	ALK kinase
−0.15	0.00	6.19E-05	b-Adrenergic Receptor kinase
−0.13	0.03	7.49E-05	Calmodulin-dependent protein kinase II
−0.22	0.01	2.71E-10	Casein kinase II
−0.04	0.05	1.88E-05	Casein Kinase II
0.52	0.10	6.32E-03	CDK5 kinase
0.07	0.05	2.88E-03	DNA dependent Protein kinase
−0.24	−0.52	5.72E-05	EGFR kinase
0.07	0.07	6.97E-07	ERK1,2 Kinase
0.04	0.05	2.15E-05	G protein-coupled receptor kinase 1
0.01	0.08	5.73E-04	Growth associated histone HI kinase
0.03	0.06	1.76E-03	GSK3 kinase
0.11	0.07	1.04E-09	GSK-3, ERK1, ERK2, CDK5
−0.12	−0.35	1.76E-03	JAK2 kinase
−0.18	0.05	1.84E-03	MAPKAPK1 kinase
0.03	0.05	4.63E-03	MAPKAPK2 kinase
−0.10	0.03	1.26E-05	PKA kinase
−0.09	0.02	8.09E-05	PKC kinase
−0.08	−0.18	1.83E-04	Src kinase

The enrichment of kinase substrate motifs with specific preferences for disorder and phosphorylation variation was calculated with the 2D annotation enrichment technique. The multiple hypotheses-corrected p-values are reported.

The class of tyrosine kinases shows a strong preference for low phosphorylation variability and structured regions, whereas the other three classes favor more disordered regions, but span a wider range of phosphorylation variation values. Also among the latter three groups higher quantitative variability is clearly associated with higher disorder. As seen in Figure 5, basophilic and acidophilic kinases occupy the regions on the graph corresponding to low phosphorylation variation, whereas proline-directed kinases are located on the right part of the graph, demonstrating their preference for more variable sites. The motif corresponding to the highest variability is the consensus motif for the proline-directed CDK5 kinase, which is in agreement with the important regulatory role of this enzyme during the cell cycle.

The proline-related class represented by two acidophilic, one basophilic and one atypical protein kinases shows preferences for intermediate level of disorderedness and phosphorylation variation properties. The Casein kinase II is characterized by various substrate recognition motifs [23], but the main differences are related to presence or absence of a proline residue preceding the phosphorylated site. These two motifs show distinct structural and variation preferences – the former type being more similar to proline-directed kinases and the latter – to non-proline ones. The occurrence of the G protein-coupled receptor kinase 1 (GRK1) near the proline-directed kinases (red triangle) can be explained analogously by the presence of a proline residue in the consensus sequence for that kinase. Interestingly, the reported consensus motifs of the MAPKAPK2 kinase (blue triangle) do not contain proline residues, however, it is still grouped together with the more variable kinase motifs. After careful examination of the amino acid composition of substrates of the MAPKAPK2 kinase in our data set we found multiple examples that contained proline within +/−6 residue window around the phospho-site. Together with our structural analysis, this suggests that this residue may play an important role in the substrate recognition. Overall, the group of proline-oriented kinases has similar preferences for disorder and phosphorylation variability as the proline-directed group. This observation also extends to the functional relevance of the member kinases to the regulation of the cell cycle. For example, the DNA-dependent protein kinase (DNA-PK) is involved in stress response and DNA repair and is known to play a role in the progression of the cell cycle [24]. Furthermore, the MAPKAPK2 kinase is involved in DNA repair processes and thus can provide an alternative to checkpoints activation [25].

Proline-directed kinases such as PLK1 are known to actively regulate the progression of the cell division cycle, thus implying that disordered regions (which are enriched for prolines) are subjected to regulation and therefore to variable phosphorylation patterns. We therefore checked if the tendency of phosphorylation variability to scale with the level of disorder persists if we control for proline-directed kinases and excluded all sites modified by such from the data set. Indeed, the effect of lower phosphorylation variability being associated with ordered regions and higher - with disordered regions remained the same.

## Discussion

Previous studies had already found a preference of phosphorylation to occur in loops or disordered regions [6],[7]. However, those studies generally did not have access to the dynamics of phosphorylation and they therefore based their analysis on the absence or presence of phosphorylation sites alone. Here, we instead made use of a large-scale quantitative phosphorylation data set to investigate a possible relation between the structural features of phosphorylation sites with their degree of regulation. This allowed us to contrast the behavior of less variable sites to those that were dynamically regulated. Our data clearly demonstrate that the propensity of phosphorylation sites to be regulated during the cell division cycle is related to the level of structural organization of the environment in which these sites reside. Furthermore, we discovered that this effect occurs in a graded manner: regions with regular structure are least likely to harbor regulated phosphorylation sites, followed by irregular regions (short loops or random coils). Note that over 90% of the sites were found within disordered structures and their high phosphorylation variability relates them to regulated phosphorylation events.

Interestingly, the sets of sites within ordered loops and disordered structures showed significant differences. It has been shown before that different flavors of disordered regions exist with regards to their lengths, amino acid composition, and the conformational transitions that they undergo upon binding [26], [27]. Liu *et al.* defined regions with no regular secondary structures (NORs) as one specific category of disordered regions. They demonstrated that NORs differ significantly from regular structured loops and argued that these might have different functional implications, a hypothesis which finds support in our study.

Functional analysis of the highly variable set of sites revealed enrichment of cell cycle-related, biosynthesis and cellular organization and localization processes (Table S1). Some examples are RNA, DNA and mRNA processing, localization and transport, regulation of gene expression and biosynthesis. Cell cycle-associated processes such as regulation of the different phases of the cycle, DNA replication and repair, telomere organization and maintenance and chromatin assembly were also strongly over-represented in the variable set of sites.

Phosphorylation is an important mechanism for regulation of a myriad of intricate processes during cell division. A detailed study of the cell cycle regulation through phosphorylation focused on functional analysis of protein groups that are up or down regulated at specific time points [2]. These were the proteins that contained sites that reached phosphorylation peaks at S or M phases. As expected, proteins involved in mitotic and cell cycle processes were shown to be maximally phosphorylated at mitosis.

Interestingly, Olsen *et al.* found proteins that regulate metabolic processes to be weakly phosphorylated during S phase and highly phosphorylated at mitosis. An explanation to this discovery is the possibly inhibitory character of phosphorylation on proteins that regulate metabolic processes, as protein synthesis and related functions tend to shut down during mitosis. Furthermore, DNA replication takes place during S phase, which rationalizes the up-regulation through phosphorylation of various proteins involved in DNA replication repair. High phosphorylation of cytokinesis-related proteins in S phase appears to play an important role in the control of the correct segregation of the two daughter cells.

The tendency of modification sites in regular structures to be less variable may be facilitated by proximal charged residues acting as stabilizers of the phosphate group. Charged flanking regions offer a suitable environment for hosting a phosphate group and allow for favorable interactions that potentially result in phosphorylation acting on a longer time scale. For instance, these favorable interactions could reduce the efficiency of phosphatases in removing a phosphate group, thereby contributing to the tendency for smaller variation in the phosphorylation level that we observe in our data. In contrast, negatively charged residues could lead to repulsion-driven conformational changes and polarization of the entire protein surface by creating clusters of negatively charged residues.

Several mechanisms that are known from literature furthermore contribute to the observed tendency for structural rather than regulatory phosphorylation sites to be present in ordered regions. Specific structural changes due to phosphorylation include stabilization of the N-termini of α-helices via favorable interactions of the added phosphate group with the helix backbone [28]. This is effected by the interaction of the phospho group with the helix dipole moment. Yet, the same modification introduced at the C-terminus would have the opposite effect [29]. The optimal stabilizing position for the phosphate group was estimated as −2 relative to the N-cap of a helix. Additionally, favorable electrostatic interactions between proximal positively charged residues (e.g. at a helix cap) and the phosphate group can enhance helix formation. The stabilizing effect of salt bridges formed between a phosphate group and a lysine side-chain has been recognized as one of the strongest possible α-helix inducers [30]. In contrast, the phosphate-guanidinium interaction leads to disruption of the local regular structure [31]. Phosphorylation has also been reported to cause conformational changes in β-sheets and disruption of β-hairpins. In those cases repulsive interactions with an aromatic tryptophan residue in the spatial vicinity of the phospho-site are observed [32]. A related question that arises from our investigation is to what extent the phosphorylation variability of a site is connected to a role in the overall structural re-arrangements of a protein. A phosphorylation event can alter the energy that is required for a conformational change [15], and thus hinder or facilitate it. Further experiments including 3D structural information or computational models are needed to increase our understanding of the interplay between structure and phosphorylation.

Multiple experimental studies show the regulatory role of modification sites that show variation in their phosphorylation patterns and lie within intrinsically disordered regions For example, the cyclin-dependent kinase inhibitor 1B (p27) is an intrinsically unstructured protein, which is multiply phosphorylated and regulates the cell cycle by inhibition of cyclin-dependent kinases (CDKs) [33]. The disorderedness of p27 plays an important role in keeping the complex formed between CDK and p27 flexible. Due to this flexibility the segment, which blocks the ATP binding site becomes exposed. This allows a tyrosine residue to become accessible for phosphorylation, upon which the space previously occupied by the inhibitor becomes available for ATP binding. Then the partially reactivated CDK phosphorylates p27 at another residue, which leads to its degradation and allows CDK to regain full activity and guide the progression through the cell cycle [34].

In another example, multiple phosphorylation sites on the transcription regulator Retinoblastoma protein (Rb) influence its ability to interact with transcription factors and other regulatory proteins. A detailed structural study reports that the different phospho-sites found within disordered regions induce distinct conformational changes and also serve different functional roles [35]. For instance, one of the modified residues decreases the affinity of Rb for binding the transcription factor E2F by reordering the pocket domain. At the same time another modified site at a loop in the pocket domain induces complete blocking of E2F binding.

We found that the set of sites with varying phosphorylation patterns was enriched in amino acids associated with disorder, specifically Pro, Gly and Ser. Interestingly the same sites were more likely to have additional modified residues in their vicinity. Phosphorylation of a protein often occurs at several distinct residues and it has been reported that modification sites tend to cluster and function in a cooperative manner [36]. Mathematical models suggest that this phenomenon leads to an increase in the sensitivity and robustness of the cellular response [37] and may promote a switch-like behavior [38]. In such a case, the exact position of a modification site in a cluster would not be a determining factor on its own, but would rather contribute to a cumulative effect. It would be worth studying how different levels of phosphorylation variability in regions with different structural organization may be implicated in the cellular regulation of the cell cycle. Multiple phosphorylation sites with highly dynamic phosphorylation patterns may be suitable for both rapid and robust response. In contrast, the robustness of the response of sites within regular regions might be achieved on a longer time scale and be related to longer lasting effects of phosphorylation.

We showed that phosphorylated residues tend to be more conserved than their equivalent non-modified residues. Conservation of phosphorylated residues has been a broadly debated issue [21], [39], but the general consensus appears to be that the overall conservation of phospho-sites is low. Even though statistically it is significantly stronger than that of the equivalent non-modified sites, the effect size is relatively small. Possible explanations include (i) loss and gain of phospho-sites at different positions in disordered regions, likely due to clusters of sites acting as functional units regardless of the exact sequence position [40] and (ii) potential silent phosphorylation events [39].

The idea that it is the cluster of phosphorylation sites that plays a functional role is becoming increasingly accepted [36], [37]. The functional roles of multiple phosphorylated residues span a wide range: (i) targeting for sub-cellular localization, (ii) targeting for degradation, (iii) control of protein-protein and protein-nucleic acid interactions (often through electrostatic effects) and (iv) enhancement of a robust and rapid response to a stimulus [1]. Furthermore, mechanisms of ‘priming’ phosphorylation are also well-known [41].

Here we showed that disordered regions harbor variable sites, which tend to be surrounded by additional phosphorylated sites. This raises the possibility that the variability of these sites is related to some of the above-described phenomena. It is known that disordered regions can facilitate a large number of interaction partners, and that multiple sites can control their association and dissociation. Given the wide range of functions of multiple phosphorylated sites in disordered regions, a larger variability in their phosphorylation patterns may provide an adequate functional mechanism to effect the desired regulation. In contrast, structural regularity imposes certain constrains on the less variable sites. The necessity of evolutionary conservation of the structure tends to prevent the accumulation of disorder-associated serine and threonine residues and a consequent change of their positions. Furthermore, the more rigid structure implies a more limited number of interactions partners. Therefore, we reason that the requirement for regulation for these sites in structured regions can be smaller.

Our data allowed us to investigate the kinase preferences of phosphorylation sites with high vs. low levels of regulation. Tyrosine kinases and kinases that require charged residues in their substrate recognition motives clearly preferred sites with smaller phosphorylation variation, whereas proline-directed kinases were clearly associated with sites that were dynamically regulated. Proline is known to be a helix and sheet breaker, due to the planarity of its side-chain. Proline lacks an NH backbone donor to form a hydrogen bond and thus disrupts the formation of regular hydrogen bond patterns, which are the basis of regular structure formation. Due to its unique stereochemistry the proline residue can adopt two different conformational states – cis and trans – and a large number of folded proteins contain both states of the residue. The intrinsic conformational changes resulting from the proline isomerization play an important role in determining the function, ligand recognition and interactions of the protein [42]. For instance, certain kinases, such as MAPKs and CDK2 preferentially modify substrates with the trans isomer [43]. Proline isomerization in a S/TP motif, where S/T is phosphorylated, can also control the opposite step – dephosphorylation, as some phosphatases appear to be conformation-specific and prefer the trans state [44]. Therefore, the preference of proline-directed kinases for sites with higher variation illustrates a connection between dynamic regulation and disordered regions.

Our results highlight the central role of proline, as a disorder-promoting residue that is also part of regulatory motifs [45]. The directing role of proline together with the multiple functions associated with disorder explain the more variable character of phosphorylation of sites with these properties that we observe in our study. Furthermore, our statistical analysis of the interplay between structure and phosphorylation variation in relation to specific kinase recognition motifs presents a new approach of describing and classifying protein kinases. We showed that the combination of both properties can be used to gain conceptual and specific insights into regulation. We were able to reproduce known relations and to identify new links between kinases, which may reflect functional dependencies emerging from common regulatory behavior and structural preferences.

In conclusion, we have related the tendency of phosphorylation sites to be dynamically regulated throughout the cell cycle with the structural features of the sites. While we have found clear relations between phosphorylation dynamics and protein structure, we are only scratching the surface of what we believe could be an exciting new area at the interface of proteomics and structural biology.

## Methods

### Computation of phosphorylation sites with high and low variability over the cell cycle

In the data set underlying our analysis [16], human HeLa S3 cells were labeled with SILAC [46], [47] to produce three different isotopic forms of lysine and arginine (light, medium and heavy). The light and heavy isotopes were synchronized in six different stages of the cell cycle, while the medium one was kept non-synchronized as a reference. Relative quantification of protein abundances (protein ratios) and/or phosphorylation (phospho-peptide ratios) were computed by taking the ratio between two cell states at each time point (*i.e.* synchronized heavy-labeled cells in S phase and non-synchronized medium-labeled cells). In order to account for the possible influence of protein abundance, changes in the phosphorylation ratios between the reference and the stimulated cells were normalized by the protein change. We mainly focused on the phosphorylation ratios as they were available for a larger number of sites compared to the absolute occupancy values.

The data set contained information about the UniProt id of the phosphorylated protein, sequence positions of phosphorylated residues, and quantitative measures of phosphorylation (normalized phosphorylation ratio) at 6 time points (i.e. cell cycle phases: G1, G1S, Early S, Late S, G2 and M). In total 1,059 proteins and 5,173 phosphorylation sites with measured phospho-ratios for each of the six time points of the cell cycle were used in the analysis.

In order to assure that the observed phenomena are not due to the properties of the chosen subset of sites, we repeated the analysis of phosphorylation variation between different structural groups with data sets containing five (5,254 sites), four (8,537 sites), and three (8,731 sites) time points only (see Text S1 for details). Although slight fluctuations were observed, the main tendencies remained stable and the conclusions did not change. Therefore, no bias in the reduced data set (*i.e.* the one containing information about all six time points) was found.

Phosphorylation variation value for each modified site was computed as the standard deviation of the phosphorylation ratios over the six time points. High variation corresponds to sites with temporal variation of phosphorylation ratios (e.g. a peak is observed in S phase), while low variation describes those sites that retain constant or slightly variable phosphorylation fold change during the cell cycle.

### Structure prediction and structural categories

The secondary structure of each site was predicted with PsiPred [18]. Each site was assigned one of three possible states: ‘H’ for α-helix (92 sites) ‘E’ for β-sheet (53 sites), and ‘C’ for random coil, turn or loop region (5,028 sites).

An intrinsic disordered state was also predicted for each site using DISOPRED [17] with standard settings. We found 498 sites to be in the ‘order’ state while the remaining 4,675 were predicted to be in the “disorder” state.

Based on a combination of secondary structure and disorder predictions, we defined three distinct structural categories for each phosphorylated site: (i) regular regions (helices and sheets in ordered regions, 145 sites), (ii) irregular regions (coils in ordered regions, 353 sites), and (iii) disordered regions (coils in disordered regions, 4,675 sites).

### Statistics

Statistical analyses were performed within the R environment [48] and using the in-house statistics work frame Perseus. The *lattice* package was utilized for comparing distributions of phosphorylation variation in different structural categories. Differences between distributions were assessed with the standard non-parametric Kolmogorov-Smirnov test. In the case of three structural categories, analysis of variance of the phosphorylation fold change was performed using the structural category as an independent variable. Data on phosphorylation site variation and structure predictions are available in the Supporting material (Table S2). Enrichment of functional Gene Ontology (GO) categories was performed with the GOrilla tool [49].

### Evolutionary analysis of phosphorylation sites

We performed conservation analysis on phosphorylated residues in ordered and disordered regions. The proteins from our data set were mapped to pre-computed EggNOG groups of orthologs [50]. We used the maximum likelihood-based *rate4site* algorithm to build phylogenetic trees from the EggNOG clusters and to compute residue-based evolutionary rates [51]. Lower evolutionary scores correspond to stronger conservation.

The ‘control’ sets of sites were defined as all serine, threonine and tyrosine residues from the phospho-proteins that were not measured to be phosphorylated in our data set with equivalent structural background (i.e. disordered and ordered as predicted by PsiPred [18]).

### Enrichment of proximal phosphorylation sites in disordered regions

We tested if disordered regions are enriched in multi-phosphorylation sites, as compared to ordered regions. We considered phosphorylation sites with at least one modified neighbor as multi phospho-sites. A neighbor residue is defined as a phosphorylated serine, threonine or tyrosine located within +/−1,2,3,4 or 5 residue-long flanking regions of a central phospho-site. For each cut-off length, we built a contingency table. Each contingency table contained the number of sites with and without neighboring phospho-sites for both ordered and disordered regions. The significance of the enrichment was estimated with the Fisher's Exact Test.

### 2D Annotation Enrichment technique

The 2D Annotation Enrichment technique [22] enables analysis of the preference of a certain group of elements (i.e. phosphorylation sites, characterized by the same consensus motif) for two numerical attributes simultaneously relative to all other elements (in our case all other phosphorylation sites). It employs a two dimensional generalization of the nonparametric two-sample test and uses the Benjamini-Hochberg method to correct for multiple hypotheses testing. We used the default settings to distinguish the statistically significant groups, corresponding to false discovery rate <0.01. We used the Human Protein Reference Database motif definitions in this analysis [23].

### Two sample logos

The difference between the amino acid content of the flanking regions of the sites with low and the sites with high phosphorylation variation was computed, assessed and visualized with the help of the *Two Sample Logo* method [19]. The highly variable set was used as the negative set. Residues significantly enriched in a certain position are shown above the horizontal line in the logo.

## Supporting Information

Table S1 **Enrichment analysis of Gene Ontology terms in the highly variable phosphorylation sites set.**
(XLS)

Table S2 **Data set of phosphorylation variations and structure predictions.**
(XLS)

Text S1 **Additional analysis.**
(DOCX)
