# Supplementary material for: Phosphorylation Variation during the Cell Cycle Scales with Structural Propensities of Proteins
Source: PLoS Comput Biol. 2013 Jan 10;9(1):e1002842. doi: 10.1371/journal.pcbi.1002842 (PMC3542066; doi:10.1371/journal.pcbi.1002842)
Supplement: Text S1 — Additional analysis. (DOCX) [file pcbi.1002842.s003.docx]

**Supplementary Notes**

**Figure S1: Length distributions of random coil and disordered regions as predicted by PsiPred and DISOPRED, respectively.**

Figure S1. Disordered regions are significantly longer (group means random coils versus disordered regions: 24.24 and 273.24 respectively, p-value 4.12E-114), suggesting a possible explanation of different phosphorylation dynamics of the sites associated with these structures.

**Enrichment of amino acids in the surrounding of phospho-sites**

In order to detect significant enrichment or depletion of amino acids in the surroundings of sites with different phosphorylation variation and to control for disorder as a possible covariate we used the composition profiling technique by Vacic *et al*.[^1^](#_ENREF_1)

As sites with dynamic phosphorylation patterns were found to be associated with disordered regions we tested amino acid enrichment versus the database of disordered proteins (DisProt). Testing disordered regions versus disordered background provided a proper negative control, and showed that serine, threonine and proline were the residues showing strongest and most significant enrichment even when controlling for the amino acid composition of disordered regions (Figure S2A, Table S3).

The compositional bias of dynamically varying phosphorylation sites was computed using the PDB25 data set compositions as background. The same results as in the previous test remained: the disorder-promoting residues serine, threonine and proline were found to be the most significantly enriched amino acids (Figure S2B, Table S4).

The same comparison with the less variable set of sites versus PDB25 resulted in the enrichment of charged residues such as aspartate, glutamate, and arginine. Additionally, serine and proline were enriched, while many hydrophobic residues were significantly depleted. (Figure S2C, Table S5).

The results of the compositional profiling technique by Vacic *et al*., support the main differences in the amino acid preferences of sites with different variation in their phosphorylation underlined by the Two Sample Logo.

**Figure S2. Enrichment of amino acids in the surrounding of modified residues with different phosphorylation variations using composition profiling.**

Figure S2. Enrichment and depletion of individual amino acids is shown. The amino acid compositions of the surrounding regions of phospho-sites with highly variable phosphorylation patterns was compared to **(A)** a background distribution of residues in the surrounding regions of non-modified serine and threonine residues in proteins from the DisProt database; **(B)** a background distribution of residues of proteins from the PDB25 database. **(C)** The distribution of amino acids in the surrounding of sites with less variable phosphorylation patterns versus a background distribution of residues of proteins from the PDB25 database.

**Control for subset selection bias**

As we limited the data by taking only sites for which quantitative information was available for all the six time points measured, we also tested for possible bias in our subsample. We performed the same analysis (Figure S3) on different subsets of the original data – taking all sites with measured phosphorylation ratios for 3 (**A)** 1.40 and 1.43 mean values in ordered and disordered regions respectively, pvalue 7.99e-6; **B)** 1.34, 1.42 and 1.43 mean values in regular, irregular and disordered regions respectively, p-value 0.08), 4 (**C)** 1.37 and 1.41 mean values, p-value 4.21e-6; **D)**1.32, 1.39 and 1.41 mean values, p-value 0.07) and 5 (**E)** 1.19 and 1.35 mean values, p-value: 6.51e-13; **F)** 1.14, 1.21 and 1.35 mean values, p-value 1.2e-4) and time points. The tendency of disordered regions to contain sites with higher variation as compared to ordered regions is preserved in all of them despite the higher noise level due to sites with missing values.

**Figure S3. Comparison of phosphorylation variation of sites with different structural background using sets of sites with a different number of measured time points.**

Figure S3. Comparison of the distributions of phosphorylation variation of sites within different structural categories. Sites with quantitative information over a different number of times points were used in each comparison. **(A, B)** all sites with quantitative measurements for at least 3 time points **(C, D)** all sites with quantitative measurements for at least 4 time points **(E, F)** all sites with quantitative measurements for at least 5 time points.

**Control for possible enrichment of disorder-related non-modified serine and threonine residues**

We compared the enrichment factors of phospho-serine and phospho-threonine residues in ordered and disordered structures as opposed to the enrichment factors of control (not found to be phosphorylated) serine and threonine residues. Modified residues showed a statistically significant preference for disordered regions (Fisher’s Exact Test odds ratio: 3.05, p-value 9.49E-151, Table S2). This test demonstrates that the presence of phospho-sites in disordered regions is not necessarily related to the enrichment of the disorder-promoting serine and threonine residues.

**Table S4. Enrichment of phosphorylated residues versus control residues in disordered regions.**

|  | pS/pT | controlS/controlT |
| --- | --- | --- |
| Disorder | 4583 | 781943 |
| Order | 447 | 232806 |

Relative enrichment of phospho-serine and phospho-threonine residues in ordered and disordered structures over control (not found to be phosphorylated) serine and threonine residues. Modified residues showed a statistically significant preference for disordered regions (Fisher’s Exact Test odds ratio: 3.05, p-value 9.49E-151).

**Table S5. Enrichment of amino acids in the surrounding environment of sites with variable phosphorylation versus the DisProt background set.**

| Amino acid | Enrichment | Significance |
| --- | --- | --- |
| A | Depleted. | P-value=0.000000 (?0.002500) |
| C | Depleted. | P-value=0.000000 (?0.002500) |
| D | Depleted. | P-value=0.000000 (?0.002500) |
| E | Depleted. | P-value=0.000000 (?0.002500) |
| F | Depleted. | P-value=0.000115 (?0.002500) |
| G | Not significant. | P-value=0.153058 (>0.002500) |
| H | Enriched. | P-value=0.001544 (?0.002500) |
| I | Depleted. | P-value=0.000000 (?0.002500) |
| K | Depleted. | P-value=0.000000 (?0.002500) |
| L | Not significant. | P-value=0.186907 (>0.002500) |
| M | Depleted. | P-value=0.000000 (?0.002500) |
| N | Depleted. | P-value=0.000000 (?0.002500) |
| P | Enriched. | P-value=0.000000 (?0.002500) |
| Q | Depleted. | P-value=0.000000 (?0.002500) |
| R | Enriched. | P-value=0.000001 (?0.002500) |
| S | Enriched. | P-value=0.000000 (?0.002500) |
| T | Enriched. | P-value=0.000000 (?0.002500) |
| V | Depleted. | P-value=0.000000 (?0.002500) |
| W | Depleted. | P-value=0.000000 (?0.002500) |
| Y | Depleted. | P-value=0.000000 (?0.002500) |

**Table S6. Enrichment of amino acids in the surrounding environment of sites with variable phosphorylation versus the PDB25 background set.**

| Amino acid | Enrichment | Significance |
| --- | --- | --- |
| A | Depleted. | P-value=0.000000 (?0.002500) |
| C | Depleted. | P-value=0.000000 (?0.002500) |
| D | Depleted. | P-value=0.000000 (?0.002500) |
| E | Depleted. | P-value=0.000000 (?0.002500) |
| F | Depleted. | P-value=0.000000 (?0.002500) |
| G | Not significant. | P-value=0.155271 (>0.002500) |
| H | Not significant. | P-value=0.018390 (>0.002500) |
| I | Depleted. | P-value=0.000000 (?0.002500) |
| K | Depleted. | P-value=0.000000 (?0.002500) |
| L | Depleted. | P-value=0.000000 (?0.002500) |
| M | Depleted. | P-value=0.000000 (?0.002500) |
| N | Depleted. | P-value=0.000000 (?0.002500) |
| P | Enriched. | P-value=0.000000 (?0.002500) |
| Q | Not significant. | P-value=0.006570 (>0.002500) |
| R | Enriched. | P-value=0.001342 (?0.002500) |
| S | Enriched. | P-value=0.000000 (?0.002500) |
| T | Enriched. | P-value=0.000000 (?0.002500) |
| V | Depleted. | P-value=0.000000 (?0.002500) |
| W | Depleted. | P-value=0.000000 (?0.002500) |
| Y | Depleted. | P-value=0.000000 (?0.002500) |

**Table S7. Enrichment of amino acids in the surrounding environment of sites with non-variable phosphorylation versus the PDB25 background set.**

| Amino acid | Enrichment | Significance |
| --- | --- | --- |
| A | Depleted. | P-value=0.000000 (?0.002500) |
| C | Depleted. | P-value=0.000000 (?0.002500) |
| D | Enriched. | P-value=0.000000 (?0.002500) |
| E | Enriched. | P-value=0.000000 (?0.002500) |
| F | Depleted. | P-value=0.000000 (?0.002500) |
| G | Depleted. | P-value=0.000201 (?0.002500) |
| H | Depleted. | P-value=0.000000 (?0.002500) |
| I | Depleted. | P-value=0.000000 (?0.002500) |
| K | Depleted. | P-value=0.000000 (?0.002500) |
| L | Depleted. | P-value=0.000000 (?0.002500) |
| M | Depleted. | P-value=0.000000 (?0.002500) |
| N | Depleted. | P-value=0.000000 (?0.002500) |
| P | Enriched. | P-value=0.000000 (?0.002500) |
| Q | Depleted. | P-value=0.000008 (?0.002500) |
| R | Enriched. | P-value=0.000000 (?0.002500) |
| S | Enriched. | P-value=0.000000 (?0.002500) |
| T | Not significant. | P-value=0.056519 (>0.002500) |
| V | Depleted. | P-value=0.000000 (?0.002500) |
| W | Depleted. | P-value=0.000000 (?0.002500) |
| Y | Depleted. | P-value=0.000000 (?0.002500) |

**References**

1. Vacic, V., Uversky, V.N., Dunker, A.K. & Lonardi, S. Composition Profiler: a tool for discovery and visualization of amino acid composition differences. *BMC Bioinformatics* 8, 211 (2007).
